# Metastasis Pattern and Survival Analysis in Primary Small Bowel Adenocarcinoma: A SEER-Based Study

**DOI:** 10.3389/fsurg.2021.759162

**Published:** 2021-12-07

**Authors:** Yanmei Gu, Haixiao Deng, Daijun Wang, Yumin Li

**Affiliations:** ^1^Department of General Surgery, Lanzhou University Second Hospital, The Second Clinical Medical College of Lanzhou University, Lanzhou, China; ^2^Key Laboratory of Digestive System Tumors of Gansu, Lanzhou, China

**Keywords:** small bowel adenocarcinoma, cancer-specific survival, overall survival, nomogram, metastasis

## Abstract

**Background:** Small bowel adenocarcinoma (SBA) is a rare gastrointestinal tumor with high malignancy. The aim of this study was to comprehensively evaluate the distant metastasis pattern and establish nomograms predicting survival for SBA.

**Methods:** From 2010 to 2015, patients diagnosed with SBA were identified based on the Surveillance, Epidemiology, and End Results (SEER) database. Kaplan–Meier survival analysis was applied to compare survival differences between metastasis patterns. Then, univariate and multivariate cox analyses were applied to screened out independent prognostic factors of cancer-specific survival (CSS) and overall survival (OS), and identify the risk factors for metastasis of SBA. To assess the discrimination and calibration of nomograms, the concordance index (C-index), calibration curves, receiver-operating characteristic curve (ROC), and decision curve analysis (DCA) were calculated.

**Results:** Kaplan–Meier curves revealed that metastasis patterns were significantly correlated with CSS (*p* < 0.001) and OS (*p* < 0.001). Then, the metastasis pattern was showed to be an independent prognostic factor of OS and CSS in patients with SBA, as well as age, grade, T stage, N stage, surgery, retrieval of regional lymph nodes, and chemotherapy. Combining these factors, we constructed prognostic nomograms, which suggested that the metastasis pattern made the greatest contribution to the survival of patients with SBA. Nomograms for OS and CSS had a C-index of 0.787 and 0.793, respectively. Calibration curves showed an excellent agreement between probability and actual observation in the training and validation cohort. Decision curve analysis also exhibited its clinical value with an improved net benefit. In addition, the models we constructed had better prognostic accuracy and clinical utility than traditional TNM staging based on C-index and ROC. Further, Cox regression analysis showed that old age, poor differentiation, N2, and not receiving chemotherapy were the risk factors for prognosis in patients with metastatic SBA.

**Conclusion:** As an independent prognostic factor, the metastasis pattern exhibited the greatest predictive effect on OS and CSS for patients with SBA. Adjuvant chemotherapy had a positive effect on the survival of patients with SBA. Nomograms for predicting 3-and 5-year OS and CSS of patients with SBA were constructed, which could identify patients with higher risk and might be superior in predicting the survival of patients with SBA than TNM staging.

## Introduction

Small bowel adenocarcinoma (SBA) often occurred in the glandular epithelium, accounting for about 36.9% in small intestinal cancer, and SBA, which mostly located in the duodenum, is the second most common histological type (1, 2). Due to the lack of specific symptoms and the narrow structure, it is difficult to diagnose SBA at the early stage (1). Moreover, the patients with SBA often need individual treatment, which depends on the original occurring position based on surgical resection, chemotherapy, radiation, and immunotherapy (3–5). Although complicated therapies were applied, the prognosis of patients with SBA with a median survival of 37% months remain poorly (6). Therefore, it is urgent to conduct an accurate prognostic analysis of patients with SBA to individualized treatment and monitoring.

Like other tumors, SBA can metastasize to the liver, lung, brain, and bone, with the liver being the dominant site of metastasis (7). Distant metastasis of SBA is one of the main causes of death. However, limited by the small sample size, few studies have analyzed and summarized the relationship between these metastasis sites and prognostic factors of multiple metastases. Therefore, it is important to identify SBA patients with early metastasis and take timely intervention measures. Patients undergoing radical surgery often die from distant metastases of the disease, suggesting the role of adjuvant chemotherapy (8).

Nomogram is a convenient and effective statistical prediction tool, widely used in cancer prognosis research (9, 10). However, the factors that affect tumor progression are complex and diverse. The nomogram can integrate and analyze various prognostic factors of individuals, simplify the classification of patients in clinical trials, and quantify the individualized outcome of patients (11, 12). Therefore, nomogram specially designed for predicting the survival of SBA is promising. This study aimed to establish the models for predicting cancer-specific survival (CSS) (*P* < 0.001) and overall survival (OS) of SBA and explore the effects of metastasis pattern on the prognosis of SBA. The Surveillance, Epidemiology, and End Results (SEER) database can provide us with corresponding data, which contains a wealth of large-scale information on tumor epidemiology, such as various clinical information and social information for specific populations (13–15).

## Materials and Methods

### Patients

**Figure 1:**
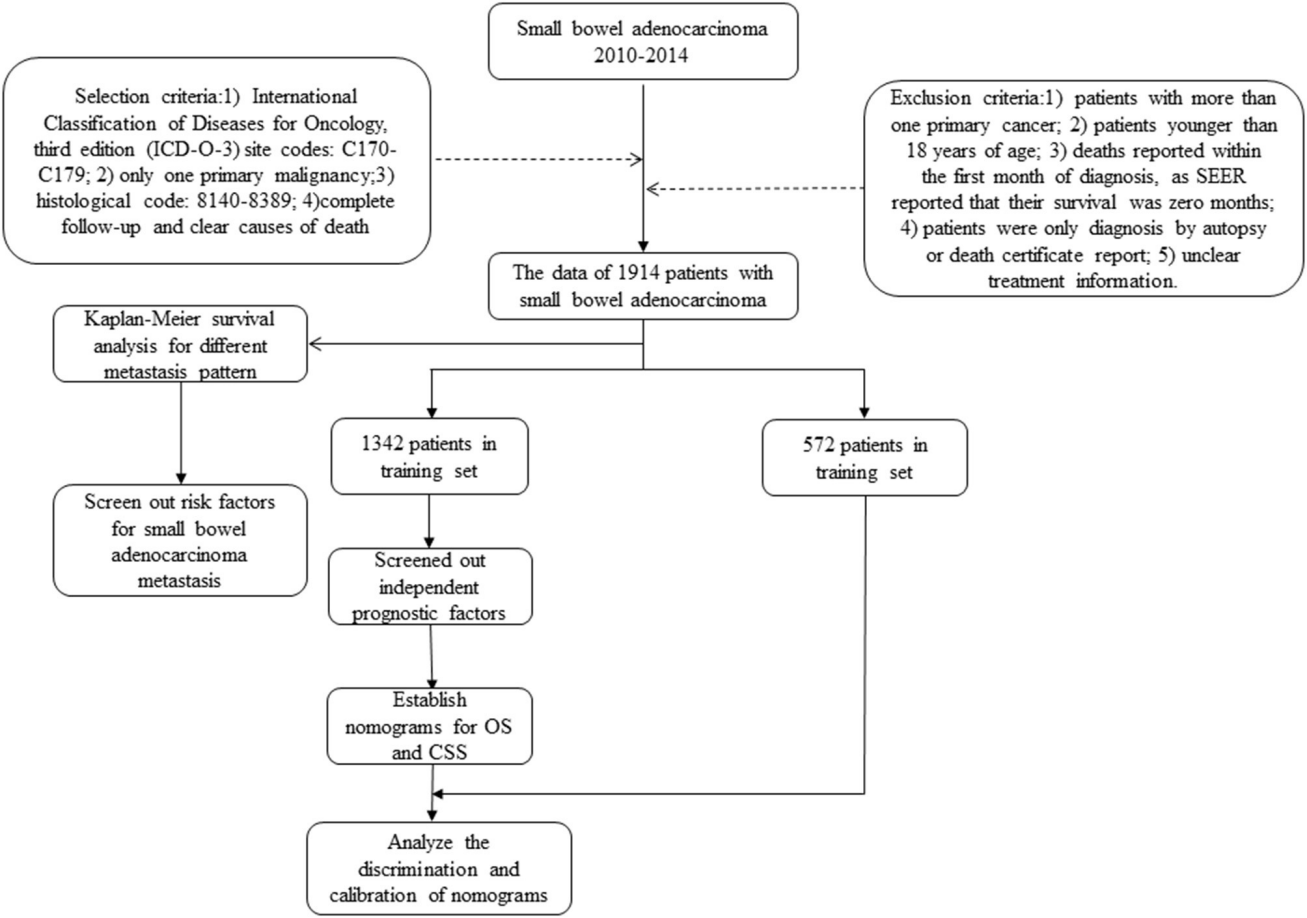
The flowchart of patients' recruitment.

The cancer incidence information collected by SEER database includes data from 18 cancer registries, covering approximately 34.6% of the US population. Data on patients diagnosed with SBA were selected from 2010 to 2014, since the information on cancer metastasis sites was available from 2010 in SEER. The inclusion criteria for patients were based on International Classification of Diseases for Oncology, third edition (ICD-O-3) site codes: C170-C179 (C170: Duodenum, C171: Jejunum, C172: Ileum, C173: Meckels diverticulum, C178: Overlapping lesion of small intestine, C179: Small intestine), only one primary malignancy, histological code: 8140-8389, complete follow-up, and clear causes of death (Supplementary Table 1). Exclusion criteria were as follows: (1) patients with more than one primary cancer; (2) patients younger than 18 years of age; (3) deaths reported within the first month of diagnosis, as SEER reported that their survival was 0 months; (4) patients were only diagnosed by autopsy or death certificate report; and (5) unclear treatment information (Figure 1). Finally, a total of 1,914 patients were included.

### Study Variables

The factors identified in the analysis included age, race, sex, marital status, primary site, histologic grade, TNM stage, surgery, chemotherapy, radiation, retrieval of reginal lymph nodes, the causes of death, and follow-up information. The endpoints of the study were OS and CSS. Overall survival is defined as the time from diagnosis to death, and CSS is the time from diagnosis to death from SBA.

### Statistical Analyses

All cases meeting the inclusion criteria were included in our study, which were divided into training cohort and validation cohort in a ratio of 7:3 by a random split method. Chi-square test was performed for the basic clinicopathological characteristics of the two cohorts. Kaplan–Meier survival analysis was used to calculate the correlation between different metastasis pattern and OS and CSS, and the log-rank test was performed to compare the significance of survival curves. All variables were evaluated by univariate and multivariate Cox regression analyses to assess the correlation between each characteristic and OS and CSS, and to identify the risk factors for metastasis of SBA. Based on the results of Cox regression analysis, nomograms were constructed for predicting 3- and 5-year OS and CSS. Concordance index (C-index) was used to evaluate the predictive ability of nomograms (16, 17). Concordance index of 0.5 indicates that the model has no predictive power, while a C-index of 1.0 indicates perfect. Receiver-operating characteristic curve (ROC) can also verify nomogram. In addition, calibration curves were drawn to assess the prognosis and actual outcome of survival. To measure the clinical application of nomogram, decision curve analysis (DCA) was applied (18, 19). SPSS software and R statistical software were used for the analysis. A *P*-value < 0.05 was considered statistically significant.

## Results

### Patient Characteristics

This study included 1,914 patients with SBA in SEER database from 2010 to 2015. Random split-sample method was used to randomly divide patients into training cohort (*n* = 1,342) and validation cohort (*n* = 572). Training cohort information was used to create nomogram, and validation cohort was applied to build external verification of nomogram. As shown in Table 1, age (*p* = 0.242), sex (*p* = 0.341), primary site (*p* = 0.089), grade (*p* = 0.865), T stage (*p* = 0.682), N stage (*p* = 0.417), metastasis status (*p* = 0.594), surgery (*p* = 0.157), retrieval of reginal lymph nodes (*p* = 0.089), radiation (*p* = 0.702), and chemotherapy (*p* = 0.112) were all factors that were similar between two groups.

**Table 1:** Patients' baseline clinicopathological characteristics.

**Characteristics**	**Training cohort**	**Validation cohort**	**Total**	***P*-value**
	**1,342 (70.1%)**	**572 (29.9%)**	**1,914 (100%)**	
**Age**				0.242
<60	454 (33.8)	177 (30.9)	631 (33.0)	
60–69	377 (28.1)	170 (29.7)	547 (28.6)	
70–79	290 (21.6)	142 (24.8)	432 (22.6)	
≥80	221 (16.5)	83 (14.5)	304 (15.9)	
**Sex**				0.341
Female	604 (45.0)	271 (47.4)	875 (45.7)	
Male	738 (55.0)	301 (52.6)	1,039 (54.3)	
**Primary site**				0.089
Duodenum	762 (56.8)	315 (55.1)	1,077 (56.3)	
Jejunum	233 (17.4)	81 (14.2)	314 (16.4)	
Ileum	199 (14.8)	105 (18.4)	304 (15.9)	
Other	148 (11.0)	71 (12.4)	219 (11.4)	
**Grade**				0.865
I/II	815 (60.7)	345 (60.3)	1,160 (60.6)	
III/IV	527 (39.3)	227 (39.7)	754 (39.4)	
**T stage**				0.682
T1/T2	223 (16.6)	87 (15.2)	310 (16.2)	
T3	425 (31.7)	194 (33.9)	619 (32.3)	
T4	555 (41.4)	228 (39.9)	783 (40.9)	
TX	139 (10.4)	63 (11.0)	202 (10.6)	
**N stage**				0.417
N0	681 (50.7)	279 (48.8)	960 (50.2)	
N1	377 (28.1)	161 (28.1)	538 (28.1)	
N2	211 (15.7)	106 (18.5)	317 (16.6)	
NX	73 (5.4)	26 (4.5)	99 (5.2)	
**Metastasis status**				0.594
No	959 (71.5)	415 (72.6)	1,374 (71.8)	
Liver metastasis	166 (12.4)	58 (10.1)	224 (11.7)	
Bone/Brain/Lung metastasis	37 (2.8)	15 (2.6)	52 (2.7)	
≥2 sites	28 (2.1)	16 (2.8)	44 (2.3)	
Other	152 (11.3)	68 (11.9)	220 (11.5)	
**Surgery**				0.157
No	368 (27.4)	139 (24.3)	507 (26.5)	
Yes	974 (72.6)	433 (75.7)	1,407 (73.5)	
**Retrieval of regional lymph nodes**				0.089
0	492 (36.7)	180 (31.5)	672 (35.1)	
1–3	118 (8.8)	52 (9.1)	170 (8.9)	
≥4	732 (54.5)	340 (59.4)	1,072 (56.0)	
**Radiation**				0.702
No	1,231 (91.7)	517 (90.4)	1,748 (91.3)	
Yes	111 (8.3)	55 (9.6)	166 (8.7)	
**Chemotherapy**				0.112
No	675 (50.3)	265 (46.3)	940 (49.1)	
Yes	667 (49.7)	307 (53.7)	974 (50.9)	

### Survival Analysis for Different Metastasis Pattern

**Figure 2:**
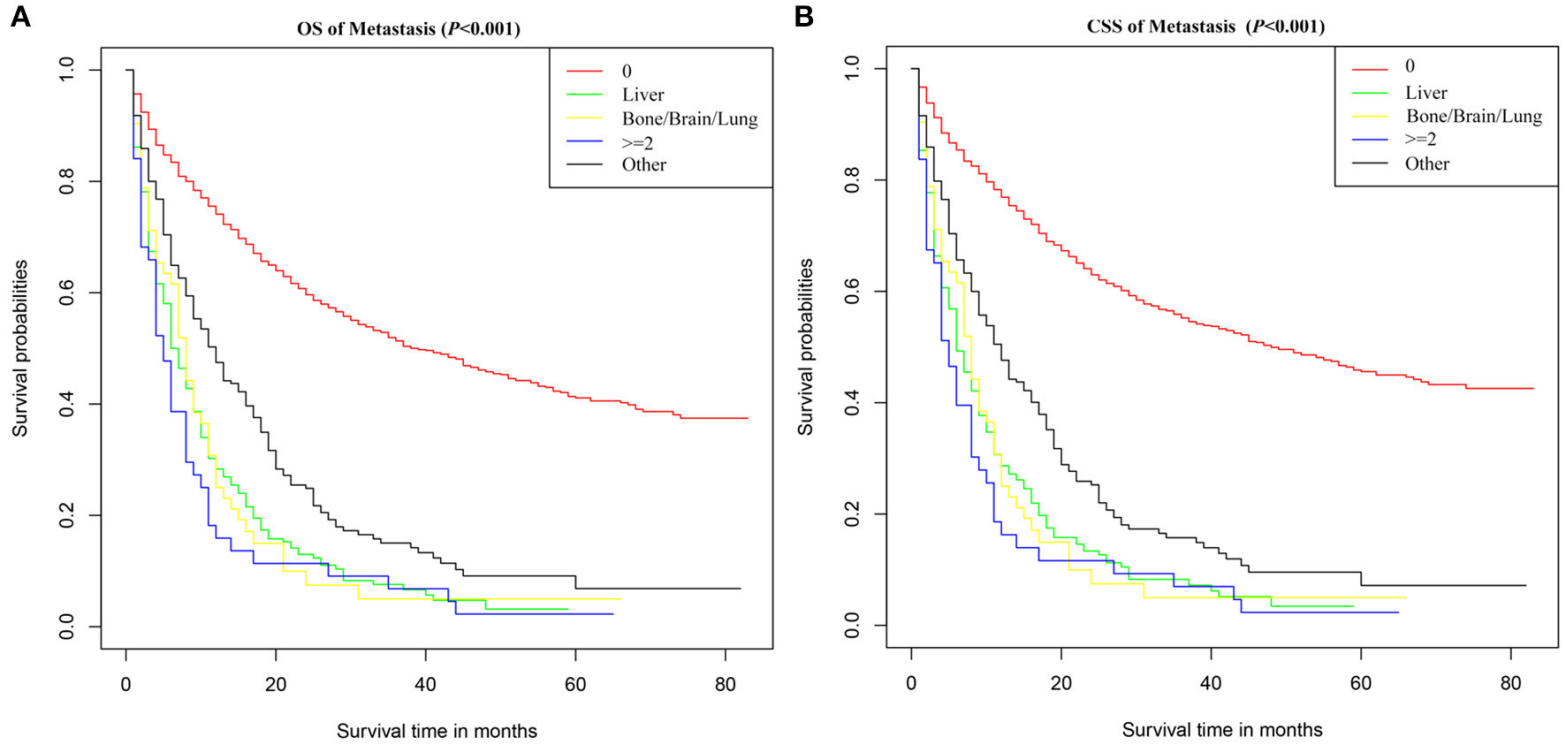
Survival curves of **(A)** OS and **(B)** CSS for SBA according to metastasis pattern. 0, no metastasis; ≥2, metastasis to at least two sites; Other, metastasis occurred, but the SEER database did not provide the site of metastasis.

In order to evaluate the impact of different metastasis patterns on OS and CSS in patients with SBA, Kaplan–Meier survival analysis was performed on all patients. As shown in Figure 2A, the difference in OS among different metastasis pattern was statistically significant (*p* < 0.001).The survival was highest for SBA patients without metastasis, followed by patients with liver and bone/brain/lung metastasis, and the worst in patients with multiple metastases. Furthermore, the Kaplan–Meier survival analysis on CSS in patients with SBA was consistent with the results of OS (Figure 2B).

### Independent Prognostic Factors for OS and CSS

**Figure 3:**
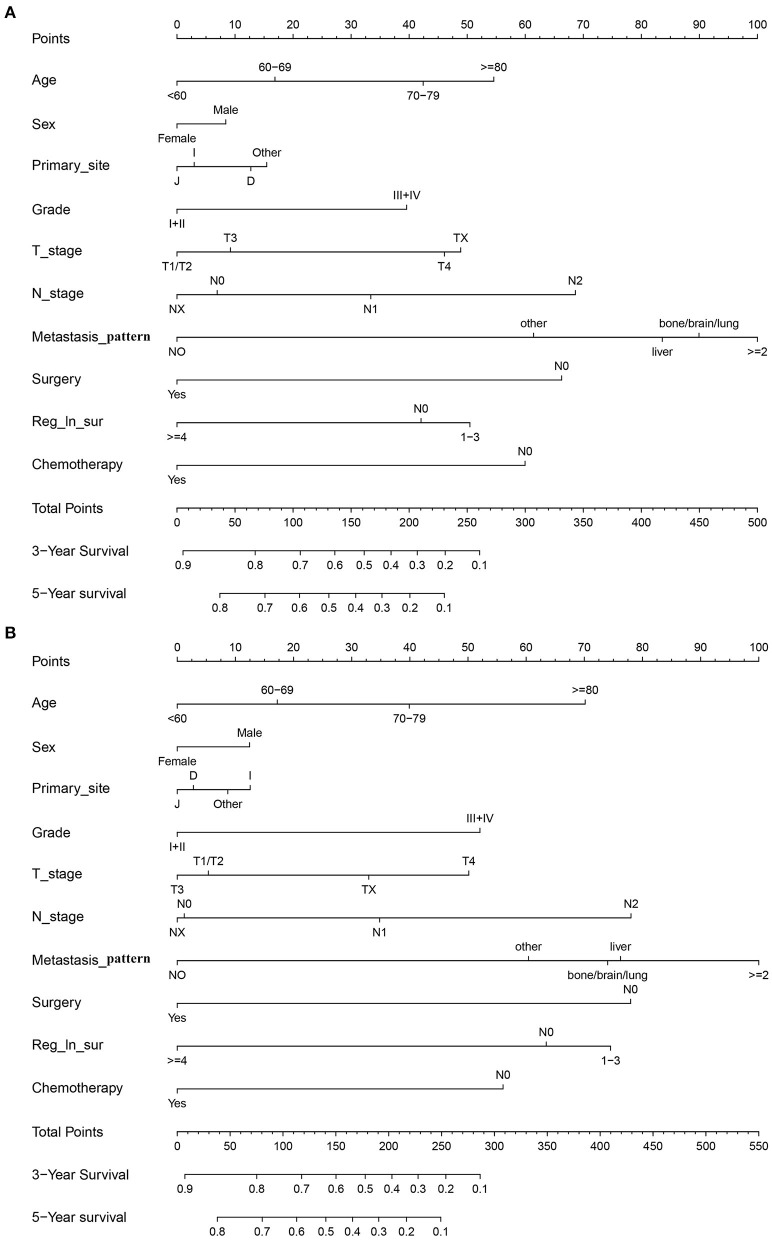
Nomograms of SBA for predicting 3- and 5-year survival **(A)** OS and **(B)** CSS.

In the training cohort, we determined that age, marital status, primary site, grade, T stage, N stage, metastasis pattern, surgery, retrieval of regional lymph nodes, and chemotherapy are related to OS and CSS by univariate Cox regression analysis (Table 2). In order to further judge whether these factors can be used as independent prognostic factors, we conducted a multivariate Cox regression analysis (Table 3). The results showed that age, grade, T stage, N stage, metastasis pattern, surgery, retrieval of regional lymph nodes, and chemotherapy were identified as independent prognostic factors for OS and CSS, which were further included in the construction of nomogram. From the clinical point of view, we also considered tumor primary site as the parameter of the nomogram (Figure 3). Nomogram showed that the metastatic pattern of SBA had the greatest impact on the prognosis, of which liver metastasis accounted for a large proportion, and surgery, N stage, and chemotherapy also made great contribution to prognosis. Age, grade, T stage, and retrieval of reginal lymph nodes showed a moderate effect on OS (Figure 3A). However, in the nomogram for CSS, the metastasis pattern was the greatest contributor, followed by surgery, N stage, and retrieval of reginal lymph nodes (Figure 3B). According to the individual characteristics of patients with SBA, the scores of each variable were added to correspond to the total score to predict the OS and CSS at 3- and 5-years. The detailed score of each variable is shown in Table 4.

**Table 2:** Univariate Cox regression analysis for overall survival and cancer-specific survival in small bowel adenocarcinoma (training cohort).

**Characteristics**	**Overall survival**		**Cancer specific survival**	
	**HR (95%CI)**	***P*-value**	**HR (95%CI)**	***P*-value**
**Age**				
<60	Reference		Reference	
60–69	1.231(1.021–1.484)	0.030	1.249(1.028–1.518)	0.025
70–79	1.739(1.437–2.104)	<0.001	1.904(1.559–2.324)	<0.001
≥80	2.434(1.994–2.971)	<0.001	2.417(1.943–3.006)	<0.001
**Sex**				
Female	Reference		Reference	
Male	1.070(0.932–1.230)	0.338	1.132(0.977–1.313)	0.099
**Primary site**				
Duodenum	Reference		Reference	
Jejunum	0.548(0.446–0.673)	<0.001	0.591(0.476–0.734)	<0.001
Ileum	0.573(0.464–0.708)	<0.001	0.652(0.528–0.806)	<0.001
Other	0.726(0.577–0.913)	0.006	0.741(0.580–0.945)	0.016
**Grade**				
I/II	Reference		Reference	
III/IV	1.757(1.529–2.019)	<0.001	1.874(1.618–2.170)	<0.001
**T stage**				
T1/T2	Reference		Reference	
T3	0.638(0.507–0.802)	<0.001	0.520(0.409–0.663)	<0.001
T4	1.328(1.083–1.629)	0.006	1.111(0.898–1.374)	0.333
TX	3.511(2.730–4.515)	<0.001	3.034(2.340–3.935)	<0.001
**N stage**				
N0	Reference		Reference	
N1	1.317(1.120–1.549)	<0.001	1.220(1.025–1.453)	0.025
N2	1.306(1.074–1.588)	0.007	1.382(1.132–1.688)	<0.002
NX	2.382(1.803–3.146)	<0.001	2.605(1.945–3.488)	<0.001
**Metastasis status**				
No	Reference		Reference	
Liver metastasis	4.354(3.600–5.266)	<0.001	4.164(3.405–5.091)	<0.001
Bone/Brain/Lung metastasis	4.490(3.172–6.354)	<0.001	4.796(3.426–6.715)	<0.001
≥2 sites	4.972(3.365–7.346)	<0.001	7.760(3.278–6.913)	<0.001
Other	2.939(2.408–3.588)	<0.001	2.815(2.283–3.471)	<0.001
**Surgery**				
No	Reference		Reference	
Yes	0.210(0.181–0.243)	<0.001	0.203(0.174–0.237)	<0.001
**Retrieval of regional lymph nodes**				
0	Reference		Reference	
1–3	0.499(0.389–0.639)	<0.001	0.473(0.365–0.615)	<0.001
≥4	0.299(0.258–0.346)	<0.001	0.274(0.234–0.320)	<0.001
**Radiation**				
No	Reference		Reference	
Yes	1.163(0.914–1.479)	0.219	1.083(0.847–1.386)	0.525
**Chemotherapy**				
No	Reference		Reference	
Yes	0.825(0.719–0.947)	0.006	0.787(0.680–0.911)	0.001

**Table 3:** Multivariate Cox regression analysis for OS and CSS in SBA patients (training cohort).

**Characteristics**	**Overall survival**		**Cancer specific survival**	
	**HR (95%CI)**	***P*-value**	**HR (95%CI)**	***P*-value**
**Age**				
<60	Reference		Reference	
60–69	1.271(1.046–1.544)	0.016	1.215(0.996–1.483)	0.056
70–79	1.783(1.453–2.190)	<0.001	1.610(1.296–2.000)	<0.001
≥80	2.024(1.607–2.550)	<0.001	2.233(1.742–2.862)	<0.001
**Sex**				
Female	Reference		Reference	
Male	1.171(1.012–1.355)	0.034	1.195(1.022–1.397)	0.026
**Primary site**				
Duodenum	Reference		Reference	
Jejunum	0.831(0.654–1.055)	0.127	0.928(0.723–1.191)	0.555
Ileum	0.884(0.695–1.124)	0.314	1.082(0.844–1.389)	0.533
Other	1.039(0.809–1.334)	0.532	1.030(0.787–1.347)	0.831
**Grade**				
I/II	Reference		Reference	
III/IV	1.612(1.395–1.863)	<0.001	1.786(1.529–2.085)	<0.001
**T stage**				
T1/T2	Reference		Reference	
T3	1.108(0.863–1.422)	0.421	0.927(0.713–1.205)	0.572
T4	1.692(1.348–2.122)	<0.001	1.608(1.272–2.033)	<0.001
TX	1.786(1.361–2.344)	<0.001	1.325(1.004–1.750)	0.047
**N stage**				
N0	Reference		Reference	
N1	1.414(1.180–1.693)	<0.001	1.445(1.191–1.754)	<0.001
N2	2.112(1.675–2.664)	<0.001	2.330(1.830–2.967)	<0.001
NX	0.930(0.689–1.256)	0.636	0.991(0.720–1.363)	0.955
**Metastasis status**				
No	Reference		Reference	
Liver metastasis	2.762(2.205–3.461)	<0.001	2.316(1.830–2.932)	<0.001
Bone/Brain/Lung metastasis	2.857(1.971–4.142)	<0.001	2.432(1.699–3.480)	<0.001
≥2 sites	3.236(2.135–4.905)	<0.001	2.970(1.995–4.420)	<0.001
Other	2.108(1.694–2.623)	<0.001	1.921(1.526–2.418)	<0.001
**Surgery**				
No	Reference		Reference	
Yes	0.445(0.335–0.591)	<0.001	0.419(0.310–0.566)	<0.001
**Retrieval of regional lymph nodes**				
0	Reference		Reference	
1–3	1.085(0.801–1.470)	0.598	1.343(0.823–1.563)	0.441
≥4	0.610(0.469–0.793)	<0.001	0.500(0.377–0.664)	<0.001
**Radiation**				
No	Reference		Reference	
Yes	1.127(0.874–1.454)	0.357	0.987(0.760–1.283)	0.923
**Chemotherapy**				
No	Reference		Reference	
Yes	0.502(0.426–0.592)	<0.001	0.547(0.459–0.651)	<0.001

**Table 4:** Detailed scores of prognostic factors in the OS and CSS nomogram.

**Characteristic**	**OS nomogram**	**CSS nomogram**
**Age**		
<60	0	0
60–69	17	17
70–79	42	40
≥80	55	70
**Sex**		
Female	0	0
Male	8	12
**Primary site**		
Duodenum	13	3
Jejunum	0	0
Ileum	3	13
Other	15	9
**Grade**		
I+II	0	0
III+IV	40	52
**T stage**		
T1+T2	0	5
T3	9	0
T4	46	50
TX	49	33
**N stage**		
N0	7	1
N1	33	35
N2	69	78
NX	0	0
**Metastasis status**		
No	0	0
Liver metastasis	84	76
Bone/Brain/Lung metastasis	90	74
≥2 sites	100	100
Other	61	60
**Surgery**		
No	66	78
Yes	0	0
**Retrieval of regional lymph nodes**		
No	42	63
1–3	50	75
≥4	0	0
**Chemotherapy**		
No	60	56
Yes	0	0

**Figure 4:**
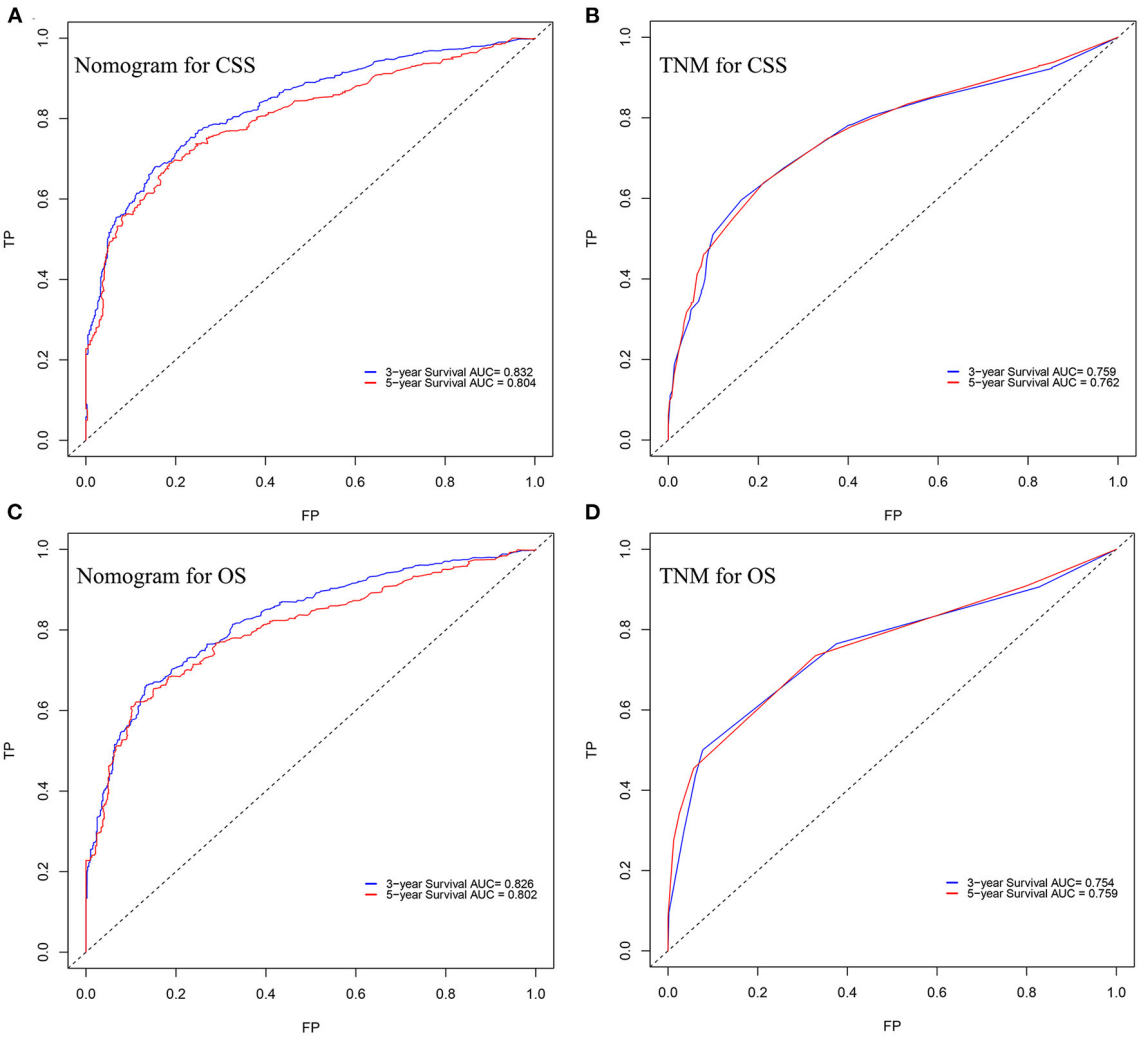
ROC curves of the nomogram for predicting **(A)** CSS and **(B)** OS at 3- and 5-year point. ROC curves of the TNM staging for predicting **(C)** CSS and **(D)** OS.

**Figure 5:**
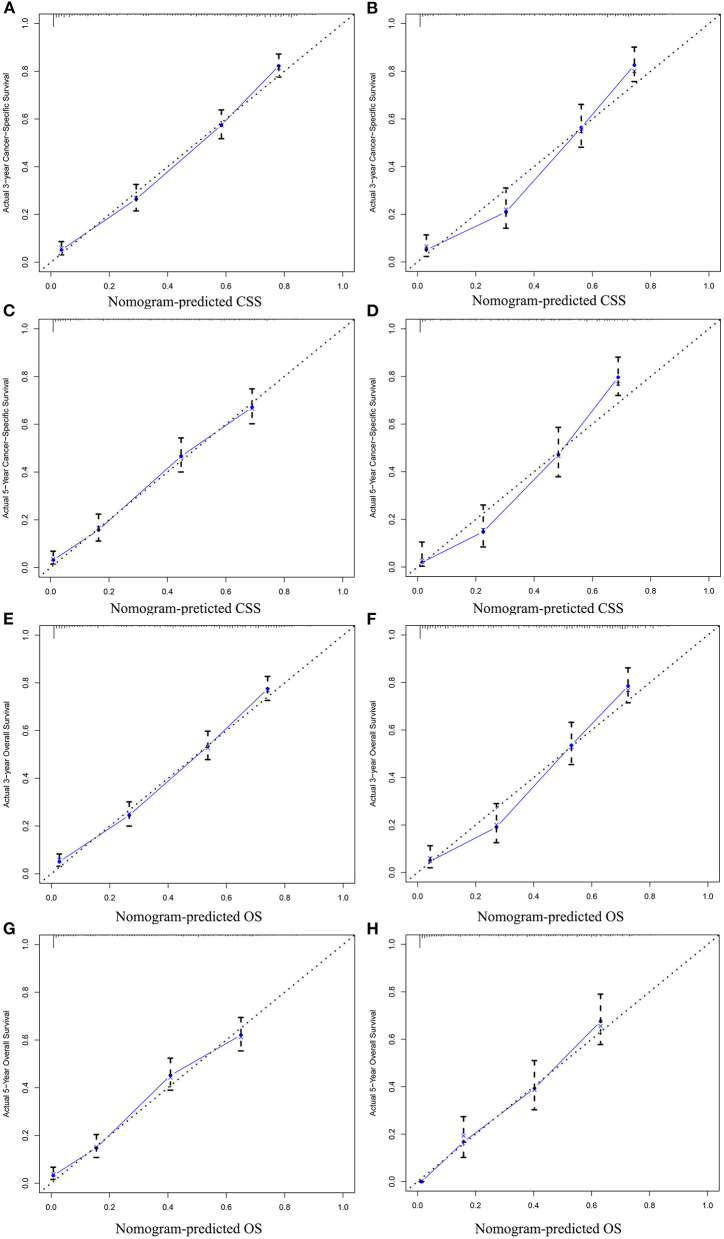
Calibration plots for predicting CSS at 3-year **(A)** training cohort and **(B)** validation cohort; calibration plots for predicting CSS at 5-year **(C)** training cohort and **(D)** validation cohort; calibration plots for predicting OS at 3-year **(E)** training cohort and **(F)** validation cohort; calibration plots for predicting OS at 5-year **(G)** training cohort, and **(H)** validation cohort.

**Figure 6:**
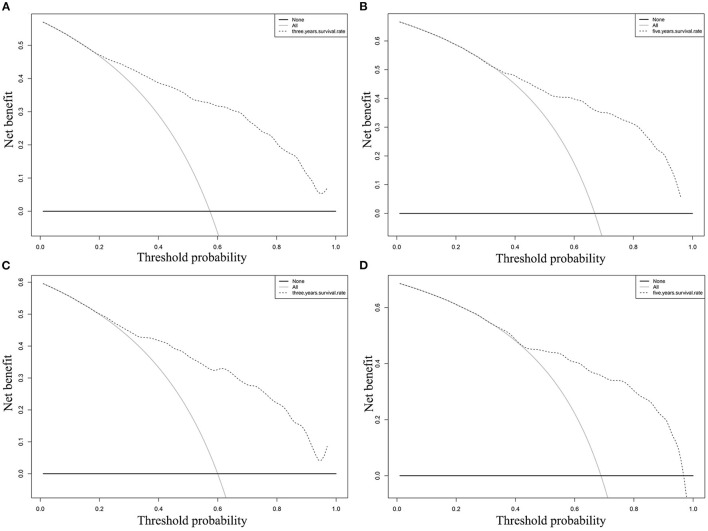
DCA of the nomogram for predicting CSS at **(A)** 3-year and **(B)** 5-year point; DCA of the nomogram for predicting OS at **(C)** 3-year and **(D)** 5-year point.

In the training cohort, the C-index of nomogram for predicting CSS and OS was 0.793 (95% CI 0.785–0.801) and 0.787 (95% CI 0.780–0.794), respectively. However, the C-index of TNM staging for CSS and OS was 0.702 (95% CI 0.692–0.712) and 0.696 (95% CI 0.687–0.705). In the validation cohort, the C-index of nomogram for CSS was 0.796 (95% CI 0.785–0.807) and that for OS was 0.762 (95% CI 0.749–0.775), which were higher than those of TNM staging [CSS: 0.718 (95% CI 0.704–0.732); OS: 0.678 (95% CI 0.663–0.693)]. The higher the C-index, the more suitable of the model for patients with SBA. By comparing the discrimination capability of nomogram with the 7th edition of AJCC TNM staging, ROC analysis showed that the nomograms we constructed also demonstrated a superior survival predictability than the 7th AJCC TNM staging system (Figure 4). Furthermore, the calibration and prediction curves of nomograms for the 3- and 5-year survival rates of patients exhibited a perfect correlation in the training cohort, indicating that nomograms for CSS and OS were well-validated (Figures 5A,C,E,G). In the validation cohort, the results showed the same reliability of the model (Figures 5B,D,F,H). Decision curve analysis indicated that the models had significantly positive net benefits within the risk of death in both cohorts, demonstrating that nomograms had good clinical value in predicting OS and CSS at 3- and 5-years (Figure 6).

### Risk Factors for SBA Distant Metastasis

We then identified the risk factors that were significantly associated with distant metastatic SBA. Among the variables studied, advanced age, tumor origin in the duodenum, poor differentiation, lack of surgical treatment at the primary site and regional lymph node dissection, and lack of radiotherapy and chemotherapy were significantly associated with OS and CSS in patients with metastatic SBA (Table 5). Variables that were statistically significant in the univariate analysis were selected for inclusion in the multivariate analysis for OS. As shown in Table 6, advanced age (*p* = 0.015), poor differentiation (*p* < 0.001), N2 stage (*p* = 0.009), absence of regional lymph node dissection (*p* = 0.007), and not receiving chemotherapy (*p* < 0.001) were significantly associated with poor prognosis in patients with metastatic SBA. Similar results were observed in the multivariate analysis for CSS.

**Table 5:** Univariate Cox regression analysis for survival in SBA patients with metastasis.

**Characteristics**	**Overall survival**		**Cancer specific survival**	
	**HR (95%CI)**	***P*-value**	**HR (95%CI)**	***P*-value**
**Age**				
<60	Reference		Reference	
60–69	1.191(0.945–1.497)	0.139	1.179(0.933–1.490)	0.167
70–79	1.517(1.189–1.934)	<0.001	1.504(1.175–1.926)	0.001
≥80	2.115(1.590–2.814)	<0.001	2.206(1.639–2.970)	<0.001
**Sex**				
Female	Reference		Reference	
Male	1.062(0.884–1.275)	0.521	1.051(0.872–1.267)	0.602
**Marital status**				
Married	Reference		Reference	
Unmarried	1.078(0.894–1.299)	0.433	1.075(0.888–1.302)	0.459
Unknown	1.292(0.835–1.998)	0.250	1.328(0.850–2.075)	0.213
**Primary site**				
Duodenum	Reference		Reference	
Jejunum	0.497(0.383–0.645)	<0.001	0.499(0.381–0.652)	<0.001
Ileum	0.584(0.440–0.774)	<0.001	0.587(0.442–0.781)	<0.001
Other	0.758(0.575–1.000)	0.051	0.742(0.558–0.988)	0.041
**Grade**				
I/II	Reference		Reference	
III/IV	1.396(1.164–1.673)	<0.001	1.391(1.156–1.675)	<0.001
**T stage**				
T1/T2	Reference		Reference	
T3	0.678(0.494–0.931)	0.016	0.669(0.485–0.924)	0.015
T4	0.724(0.552–0.950)	0.020	0.721(0.548–0.949)	0.019
TX	1.300(0.966–1.750)	0.084	1.323(0.979–1.788)	0.068
**N stage**				
N0	Reference		Reference	
N1	0.790(0.636–0.980)	0.032	0.800(0.642–0.998)	0.048
N2	0.829(0.643–1.068)	0.146	0.854(0.660–1.107)	0.233
NX	1.038(0.752–1.434)	0.820	1.058(0.760–1.472)	0.738
**Surgery**				
No	Reference		Reference	
Yes	0.491(0.408–0.591)	<0.001	0.487(0.4026–0.588)	<0.001
**Retrieval of regional lymph nodes**				
0	Reference		Reference	
1–3	0.621(0.450–0.858)	0.004	0.608(0.435–0.850)	0.004
≥4	0.509(0.412–0.628)	<0.001	0.505(0.407–0.627)	<0.001
**Radiation**				
No	Reference		Reference	
Yes	1.664(1.195–2.317)	0.003	1.633(1.167–2.285)	0.004
**Chemotherapy**				
No	Reference		Reference	
Yes	0.477(0.395–0.577)	<0.001	0.462(0.381–0.561)	<0.001

**Table 6:** Multivariate Cox regression analysis for survival in SBA patients with metastasis.

**Characteristics**	**Overall survival**		**Cancer specific survival**	
	**HR (95%CI)**	***P*-value**	**HR (95%CI)**	***P*-value**
**Age**				
<60	Reference		Reference	
60–69	1.221(0.964–1.545)	0.097	1.214(0.955–1.544)	0.113
70–79	1.261(0.979–1.626)	0.073	1.236(0.954–1.601)	0.109
≥80	1.462(1.076–1.987)	0.015	1.512(1.098–2.082)	0.011
**Primary site**				
Duodenum	Reference		Reference	
Jejunum	0.759(0.552–1.044)	0.09	0.802(0.575–1.118)	0.192
Ileum	0.865(0.600–1.247)	0.436	0.932(0.641–1.356)	0.714
Other	1.187(0.866–1.628)	0.286	1.202(0.868–1.664)	0.267
**Grade**				
I/II	Reference		Reference	
III/IV	1.600(1.325–1.932)	<0.001	1.564(1.290–1.897)	<0.001
**T stage**				
T1/T2	Reference		Reference	
T3	0.999(0.709–1.410)	0.999	0.992(0.699–1.408)	0.965
T4	0.925(0.688–1.244)	0.604	0.928(0.687–1.254)	0.626
TX	1.179(0.869–1.600)	0.291	1.211(0.889–1.649)	0.224
**N stage**				
N0	Reference		Reference	
N1	1.059(0.843–1.331)	0.623	1.100(0.869–1.392)	0.430
N2	1.517(1.108–2.078)	0.009	1.576(1.145–2.170)	0.005
NX	0.911(0.650–1.278)	0.590	0.962(0.680–1.361)	0.825
**Surgery**				
No	Reference		Reference	
Yes	0.708(0.495–1.014)	0.059	0.714(0.495–1.029)	0.071
**Retrieval of regional lymph nodes**				
0	Reference		Reference	
1–3	0.991(0.673–1.460)	0.963	0.976(0.655–1.455)	0.906
≥4	0.601(0.416–0.869)	0.007	0.567(0.389–0.826)	0.003
**Radiation**				
No	Reference		Reference	
Yes	1.156(0.814–1.640)	0.419	1.133(0.794–1.615)	0.492
**Chemotherapy**				
No	Reference		Reference	
Yes	0.456(0.371–0.561)	<0.001	0.442(0.357–0.546)	<0.001

## Discussion

The malignant small bowel tumor is a rare gastrointestinal tumor, accounting for about 5% of all gastrointestinal tumors (20). Small bowel adenocarcinoma is one of the most common histological subtypes, which has been found to have a very bad prognosis (21). The AJCC TNM staging system neglects taking some significant factors into consider, though it is applied to predict the survival of patients with SBA. Although recent study has constructed a prognostic model for SBA, this study ignores the impact of adjuvant chemotherapy, radiotherapy, lymph node dissection, and tumor metastasis patterns on the survival of patients with SBA (22). Compared with previous prognostic models, our study established the most comprehensive nomogram for patients with SBA. Concordance index, calibration curves, ROC, and DCA all proved good predictive ability and clinical application of nomograms. The new model showed more predictability than the 7th edition of AJCC TNM staging system both in the training test and in the validation set. We further identify the risk factors of patients with metastatic SBA.

Age, tumor grade, T stage, and N stage were shown to be independent prognostic factors for OS and CSS in patients with SBA, consistent with previous studies (6, 20, 23, 24). Old age was one of the factors for the poor prognosis of many malignant tumors, which may be attributed to the decline of the patient's body resistance. Poorly differentiated or undifferentiated of tumors often led to the malignant progression; thus, it was the factor affecting the survival. In addition, our model indicated that T4 and N2 were the important risk factors for patients with SBA. In a prospective study based on 347 patients with SBA, T4 (*p* = 0.001) was correlated with a higher risk of death, which was an independent prognostic factor for OS in patients with SBA (25). Another retrospective study also emphasized T4 as an independent prognostic factor (26). Previous retrospective studies have shown that lymph node involvement has adverse effects on patients with SBA (7). It should be noted that surgery, number of lymph node dissection, and chemotherapy have strong prognostic values for patients with CSS and OS, demonstrating the positive effect of surgical treatment and systemic chemotherapy on patients with SBA. At present, surgery remains the main treatment option for patients with SBA, while radical surgical resection and adequate lymph node dissection are important means to improve the prognosis of patients. In many past studies, adjuvant chemotherapy could significantly improve OS and disease-free survival (DFS) (27, 28). Another retrospective study pointed out that adjuvant chemotherapy did not improve the OS and DFS of patients with SBA after surgery (29). In our study, patients receiving chemotherapy had a lower risk of survival (HR = 0.502, *P* < 0.001), suggesting that adjuvant chemotherapy has a positive effect on improving the prognosis of patients with SBA. However, radiotherapy had no significant effect on the prognosis of SBA based on the Cox analysis. For solid tumors, there were still uncertainties in dose selection, time, and toxicity of radiotherapy (30). Due to the rare incidence of small intestinal adenocarcinoma, the analysis of the effect of therapies was limited by the small sample size, so the effect of radiation on patients with SBA was still controversial (21, 23, 26, 28). In addition, studies have shown that the primary tumor site was an independent prognostic factor in patients with SBA (31, 32). This may be attributed to the fact that the early diagnosis rate of SBA that originated in the duodenum was higher than that of jejunum and ileum tumors, and they were treated relatively early. In univariate Cox analysis, the primary tumor site was significant, but multivariate regression analysis suggested negative results.

Among the included variables, the metastatic pattern was a significant independent prognostic factor and was positively correlated with the risk of death of the patient. The results of survival analysis were consistent. Small bowel adenocarcinoma patients with more than two metastasis sites had a significantly negative prognosis in this model. The study showed poor OS in patients with metastatic SBA, consistent with our study (33). Based on the results of univariate Cox analysis, multivariate Cox regression again emphasized that poorly differentiated, N2, and absence of chemotherapy were negatively associated with OS and CSS in metastatic SBA patients. A systematic review of seven prospective studies revealed that adjuvant chemotherapy was effective for unresectable or metastatic SBA (34). In a retrospective analysis of patients with metastatic SBA, patients who received palliative chemotherapy had a median OS of 9.3 months, significantly better than those who did not receive chemotherapy (35). These studies suggested that adjuvant chemotherapy had a certain benefit in improving the prognosis of metastatic SBA. However, the current SBA chemotherapy regimens mostly refer to other gastrointestinal malignancies, and the efficacy of different chemotherapy regimens varies greatly. In a multicenter retrospective analysis, oxaliplatin-based chemotherapy regimens were found to significantly improve the prognosis of patients with metastatic SBA compared with cisplatin (36). Limitations of our study were the lack of detailed protocols for chemotherapy and targeted therapy regimens and the absence of a history of Crohn's disease.

In summary, we have established and verified two clinically effective nomograms predicting OS and CSS for patients with primary SBA at 3- and 5-years, based on a large number of people. The prognosis of metastatic SBA is poor. Advanced age, poor differentiation, and N2 are the risk factors for metastatic SBA. Adjuvant chemotherapy can improve the prognosis of metastatic SBA to a certain extent.

## Data Availability Statement

The original contributions presented in the study are included in the article/Supplementary Material, further inquiries can be directed to the corresponding author/s.

## Author Contributions

YG, HD, and YL designed the research and wrote the manuscript. YG and HD processed and analyzed the data. DW assisted in the data processing. YL conceived of the study and coordinated the study. All authors gave final approval for publication.

## Conflict of Interest

The authors declare that the research was conducted in the absence of any commercial or financial relationships that could be construed as a potential conflict of interest.

## Publisher's Note

All claims expressed in this article are solely those of the authors and do not necessarily represent those of their affiliated organizations, or those of the publisher, the editors and the reviewers. Any product that may be evaluated in this article, or claim that may be made by its manufacturer, is not guaranteed or endorsed by the publisher.

## References

[1] Benson AB Venook AP Al-Hawary MM Arain MA Chen YJ Ciombor KK. Small bowel adenocarcinoma, version 1.2020, NCCN clinical practice guidelines in oncology. J Natl Compr Canc Netw. (2019) 17:1109–33. 10.6004/jnccn.2019.0043 31487687 PMC10191182

[2] Giuffrida P Vanoli A Di Sabatino A. Survival in Crohn's disease-associated small bowel adenocarcinoma. Gut. (2021) 70:997–8. 10.1136/gutjnl-2020-322364 32709612

[3] Aparicio T Svrcek M Henriques J Afchain P Lièvre A Tougeron D. Panel gene profiling of small bowel adenocarcinoma: results from the NADEGE prospective cohort. Int J Cancer. (2021) 148:1731–42. 10.1002/ijc.33392 33186471

[4] Latham A Shia J Patel Z Reidy-Lagunes DL Segal NH Yaeger R. Characterization and clinical outcomes of DNA mismatch repair-deficient small bowel adenocarcinoma. Clin Cancer Res. (2021) 27:1429–37. 10.1158/1078-0432.CCR-20-2892 33199489 PMC7925361

[5] Pedersen KS Foster NR Overman MJ Boland PM Kim SS Arrambide KA. ZEBRA: a multicenter phase II study of pembrolizumab in patients with advanced small-bowel adenocarcinoma. Clin Cancer Res. (2021) 27:3641–8. 10.1158/1078-0432.CCR-21-0159 33883178

[6] Sakae H Kanzaki H Nasu J Akimoto Y Matsueda K Yoshioka M. The characteristics and outcomes of small bowel adenocarcinoma: a multicentre retrospective observational study. Br J Cancer. (2017) 117:1607–13. 10.1038/bjc.2017.338 28982111 PMC5729438

[7] Dabaja BS Suki D Pro B Bonnen M Ajani J. Adenocarcinoma of the small bowel: presentation, prognostic factors, and outcome of 217 patients. Cancer. (2004) 101:518–26. 10.1002/cncr.20404 15274064

[8] Halfdanarson TR McWilliams RR Donohue JH Quevedo JF. A single-institution experience with 491 cases of small bowel adenocarcinoma. Am J Surg. (2010) 199:797–803. 10.1016/j.amjsurg.2009.05.037 20609724

[9] Wang S Yang L Ci B Maclean M Gerber DE Xiao G. Development and validation of a nomogram prognostic model for SCLC patients. J Thorac Oncol. (2018) 13:1338–48. 10.1016/j.jtho.2018.05.037 29902534 PMC7678404

[10] Liao Y Yin G Fan X. The positive lymph node ratio predicts survival in T(1-4)N(1-3)M(0) non-small cell lung cancer: a nomogram using the SEER database. Front Oncol. (2020) 10:1356. 10.3389/fonc.2020.01356 32903785 PMC7438846

[11] He Y Zhu Z Chen Y Chen F Wang Y Ouyang C. Development and validation of a novel diagnostic nomogram to differentiate between intestinal tuberculosis and Crohn's disease: a 6-year prospective multicenter study. Am J Gastroenterol. (2019) 114:490–9. 10.14309/ajg.0000000000000064 30741735

[12] Tanadini-Lang S Rieber J Filippi AR Fode MM Streblow J Adebahr S. Nomogram based overall survival prediction in stereotactic body radiotherapy for oligo-metastatic lung disease. Radiother Oncol. (2017) 123:182–8. 10.1016/j.radonc.2017.01.003 28169042

[13] Liang W He J Shen Y Shen J He Q Zhang J. Impact of examined lymph node count on precise staging and long-term survival of resected non-small-cell lung cancer: a population study of the US SEER database and a Chinese multi-institutional registry. J Clin Oncol. (2017) 35:1162–70. 10.1200/JCO.2016.67.5140 28029318 PMC5455598

[14] Doll KM Rademaker A Sosa JA. Practical guide to surgical data sets: surveillance, epidemiology, and end results (SEER) database. JAMA Surg. (2018) 153:588–9. 10.1001/jamasurg.2018.0501 29617544

[15] Abdel-Rahman O. Challenging a dogma: five-year survival does not equal cure in all colorectal cancer patients. Expert Rev Anticancer Ther. (2018) 18:187–92. 10.1080/14737140.2018.1409625 29168934

[16] Moskowitz CS. Using free-response receiver operating characteristic curves to assess the accuracy of machine diagnosis of cancer. Jama. (2017) 318:2250–1. 10.1001/jama.2017.18686 29234793

[17] Harrell FE Jr Lee KL Mark DB. Multivariable prognostic models: issues in developing models, evaluating assumptions and adequacy, and measuring and reducing errors. Stat Med. (1996) 15:361–87. 10.1002/(SICI)1097-0258(19960229)15:4<361::AID-SIM168>3.0.CO;2-4 8668867

[18] Zhang Z Rousson V Lee WC Ferdynus C Chen M Qian X. Decision curve analysis: a technical note. Ann Transl Med. (2018) 6:308. 10.21037/atm.2018.07.02 30211196 PMC6123195

[19] Van Calster B Wynants L Verbeek JFM Verbakel JY Christodoulou E Vickers AJ. Reporting and interpreting decision curve analysis: a guide for investigators. Eur Urol. (2018) 74:796–804. 10.1016/j.eururo.2018.08.038 30241973 PMC6261531

[20] Aparicio T Zaanan A Svrcek M Laurent-Puig P Carrere N Manfredi S. Small bowel adenocarcinoma: epidemiology, risk factors, diagnosis and treatment. Dig Liver Dis. (2014) 46:97–104. 10.1016/j.dld.2013.04.013 23796552

[21] Delaunoit T Neczyporenko F Limburg PJ Erlichman C. Small bowel adenocarcinoma: a rare but aggressive disease. Clin Colorectal Cancer. (2004) 4:241–8; discussion 249–51. 10.3816/CCC.2004.n.023 15555205

[22] Zheng Z Zhou X Zhang J Zhao B Chen C Liu X. Nomograms predict survival of patients with small bowel adenocarcinoma: a SEER-based study. Int J Clin Oncol. (2021) 26:387–98. 10.1007/s10147-020-01813-8 33113018

[23] Koo DH Yun SC Hong YS Ryu MH Lee JL Chang HM. Systemic chemotherapy for treatment of advanced small bowel adenocarcinoma with prognostic factor analysis: retrospective study. BMC Cancer. (2011) 11:205. 10.1186/1471-2407-11-205 21619586 PMC3125281

[24] Hong SH Koh YH Rho SY Byun JH Oh ST Im KW. Primary adenocarcinoma of the small intestine: presentation, prognostic factors and clinical outcome. Jpn J Clin Oncol. (2009) 39:54–61. 10.1093/jjco/hyn122 18997182

[25] Aparicio T Henriques J Manfredi S Tougeron D Bouché O Pezet D. Small bowel adenocarcinoma: results from a nationwide prospective ARCAD-NADEGE cohort study of 347 patients. Int J Cancer. (2020) 147:967–77. 10.1002/ijc.32860 31912484

[26] Huffman BM Jin Z Yadav S Patel S Nagorney DM Truty MJ. Novel prognostic factors in resected small bowel adenocarcinoma. Clin Colorectal Cancer. (2019) 18:218–25. 10.1016/j.clcc.2019.05.002 31178274

[27] Li N Shen W Deng W Yang H Ma Y Bie L. Clinical features and the efficacy of adjuvant chemotherapy in resectable small bowel adenocarcinoma: a single-center, long-term analysis. Ann Transl Med. (2020) 8:949. 10.21037/atm-20-1503 32953749 PMC7475383

[28] Ecker BL McMillan MT Datta J Mamtani R Giantonio BJ Dempsey DT. Efficacy of adjuvant chemotherapy for small bowel adenocarcinoma: a propensity score-matched analysis. Cancer. (2016) 122:693–701. 10.1002/cncr.29840 26717303

[29] Aydin D Sendur MA Kefeli U Unal OU Tastekin D Akyol M. Evaluation of prognostic factors and adjuvant chemotherapy in patients with small bowel adenocarcinoma who underwent curative resection. Clin Colorectal Cancer. (2017) 16:220–7. 10.1016/j.clcc.2016.08.002 27670893

[30] Tzeng CW Fiveash JB Heslin MJ. Radiation therapy for retroperitoneal sarcoma. Expert Rev Anticancer Ther. (2006) 6:1251–60. 10.1586/14737140.6.8.1251 16925491

[31] Wilhelm A Galata C Beutner U Schmied BM Warschkow R Steffen T. Duodenal localization is a negative predictor of survival after small bowel adenocarcinoma resection: a population-based, propensity score-matched analysis. J Surg Oncol. (2018) 117:397–408. 10.1002/jso.24877 29044591

[32] Falcone R Romiti A Filetti M Roberto M Righini R Botticelli A. Impact of tumor site on the prognosis of small bowel adenocarcinoma. Tumori. (2019) 105:524–8. 10.1177/0300891619839297 30935289

[33] Rompteaux P Gagnière J Gornet JM Coriat R Baumgaertner I Lecomte T. Resection of small bowel adenocarcinoma metastases: results of the ARCAD-NADEGE cohort study. Eur J Surg Oncol. (2019) 45:331–5. 10.1016/j.ejso.2018.11.012 30501999

[34] Nishikawa Y Hoshino N Horimatsu T Funakoshi T Hida K Sakai Y. Chemotherapy for patients with unresectable or metastatic small bowel adenocarcinoma: a systematic review. Int J Clin Oncol. (2020) 25:1441–9. 10.1007/s10147-020-01703-z 32448950

[35] Legué LM Bernards N Lemmens VE de Hingh IH Creemers GJ van Erning FN. Palliative chemotherapy for patients with synchronous metastases of small-bowel adenocarcinoma: a reflection of daily practice. Unit Eur Gastroenterol J. (2019) 7:1380–8. 10.1177/2050640619858211 31839964 PMC6894007

[36] Zaanan A Costes L Gauthier M Malka D Locher C Mitry E. Chemotherapy of advanced small-bowel adenocarcinoma: a multicenter AGEO study. Ann Oncol. (2010) 21:1786–93. 10.1093/annonc/mdq038 20223786

