# Supplementary material for: Metastasis Pattern and Survival Analysis in Primary Small Bowel Adenocarcinoma: A SEER-Based Study
Source: Front Surg. 2021 Dec 7;8:759162. doi: 10.3389/fsurg.2021.759162 (PMC8691381; doi:10.3389/fsurg.2021.759162)
Supplement: Supplementary file 1 [file Table_1.docx]

| **Histological code** | **Description** |
| --- | --- |
| 8140 | Adenocarcinoma |
| 8143 | Superficial spreading adenocarcinoma |
| 8144 | Adenocarcinoma, intestinal type |
| 8145 | Carcinoma, diffuse type |
| 8210 | Adenocarcinoma in adenomatous polyp |
| 8211 | Tubular adenocarcinoma |
| 8220 | Adenocarcinoma in adenomatous polyposis coli |
| 8221 | Adenocarcinoma in multiple adenomatous polyps |
| 8243 | Goblet cell carcinoid |
| 8244 | Mixed adenoneuroendocrine carcinoma |
| 8245 | Adenocarcinoid tumor |
| 8255 | Adenocarcinoma with mixed subtypes |
| 8261 | Adenocarcinoma in villous adenoma |
| 8262 | Villous adenocarcinoma |
| 8263 | Adenocarcinoma in tubulovillous adenoma |
| 8380 | Endometrioid carcinoma |

**Table S1**: The interpretation of histological code
